# Primary and middle-school children’s drawings of the lockdown in Italy

**DOI:** 10.3389/fpsyg.2022.982654

**Published:** 2022-10-18

**Authors:** Michele Capurso, Livia Buratta, Claudia Mazzeschi

**Affiliations:** Department of Philosophy, Social Sciences and Education, University of Perugia, Perugia, Italy

**Keywords:** children, drawings, COVID-19, lockdown, experience, child development

## Abstract

This retrospective-descriptive study investigated how primary and middle-school children perceived the first COVID-19 lockdown in Italy (March–May 2020) as manifested in their drawings. Once school restarted after the first COVID-19 wave, and as part of a structured school re-entry program run in their class in September 2020, 900 Italian children aged 7–13 were asked to draw a moment of their life during the lockdown. The drawings were coded and quantitatively and qualitatively analyzed; several pictorial examples are illustrated in this article. Most children used colorful and full-body representations of the self, but in almost half of the pictures drawn by older students, the self was either missing or represented without the face visible. Most children drew the interior of their houses, and the outside world was completely invisible in over half of the pictures. The most represented activities among younger students were playing or sport, followed by screentime or technology-use. Domestic routines and distance learning were also depicted. Most children, but predominantly girls, drew characters showing emotional cohesion clues, and more younger pupils and girls depicted contentment as their main emotion. Conflicting emotions were virtually non-existent. Our data suggest that children coped with the lockdown through play, screen, and technology use. The high incidence of the missing self-representation in preadolescents could indicate how the enforced loneliness and lack of direct physical contact with others impacted their perception of the self. The findings presented here deepen our knowledge of the dynamics connected to the effects of the COVID crisis on children and young people and show how drawings can provide a valuable window into children’s emotions and perceptions.

## Introduction

On 4 March 2020, Italy declared its first national lockdown to respond to the COVID-19 pandemic (Presidenza del Consiglio dei Ministri, 2020). Consequently, nine million Italian children were confined in their homes and did not see their teachers or classmates until the following September. Alongside the isolation from their peers and teachers, outdoor activities were also limited or banned. These youngsters had to cope with psychological stressors such as loneliness, insecurity, and instability in their daily routines (Samji et al., 2022; Viner et al., 2022). Fear, loneliness, and boredom permeated the pandemic experiences and daily lives of children across the country (Cellini et al., 2021). International research shows that the lockdown affected the psychological state of approximately 79.4% of children (Panda et al., 2020), who showed signs of increasing anxiety, depression, irritability, and inattention. Additionally, approximately 25% of children developed a substantial fear of the virus, and about 33% experienced trouble sleeping. Such effects were mediated by a composite network of factors, including culture (Furlong and Finnie, 2020), socioeconomic status (Save the Children Italia, 2020), age, and gender (Davico et al., 2021). However, despite the extent and magnitude of the phenomenon, children also responded to the lockdown with resilience and adaptability. Coping factors emerged as products of social-cultural and geographical peculiarities (especially in terms of lifestyle, education, participation in family life, communication, access and use of personal and open spaces, possibility to use technologies actively and creatively; Cortés-Morales et al., 2021). Stoecklin et al. (2021) report that during the lockdown, some children felt free to use their time as they wished and took this opportunity to spend more time with their families, start new hobbies, take care of themselves, and adopt a slower pace of life.

## Children’s drawings as a representation of reality

One way in which people communicate their experiences, perceptions, or attitudes toward an event is through freehand drawing (Walker, 2007). Drawing an experience has several advantages over other verbal methods in children. It helps them communicate what is not easily put into words, extending their voice and participation, especially for those who are more hesitant to speak or share ideas (Bland, 2018). Through drawings, children graphically and emotionally represent what they feel to be significant according to their understanding and interpretation of the event (Wang and Brown, 2019). Additionally, drawing can be a valuable tool in problem-solving (Soundy and Drucker, 2009).

For children, drawing is one of many developmentally appropriate narrative activities that can take different forms (e.g., playing, story-telling, writing, and performing; Kerry-Moran and Aerila, 2019). Narratives are a way to recount one or more events organized in an order assumed to show the sharer’s perspective of the episode (Bruner, 1986). As such, for the child, drawing can be a meaning-making, purposeful activity (Einarsdottir et al., 2009). Drawings provide fundamental intra- and inter-personal universes in which children make sense of their relationship between themselves and the world around them (Capurso et al., 2021b). These characteristics make drawings a rich, creative, and colorful source of information for researchers.

The connection between the different narrative forms and reality is complex, especially in children. First is the issue of the intricate intermingling between sensation, perception, and imagination when recalling events (Johnson and Foley, 1984; Cavallina et al., 2018). Next, from a phenomenological perspective, multiple realities connect the events with their narratives. For example, the anthropologist Bruner (1984) differentiates between the “life as lived” (flow of events that touch a person’s life), the “life as experienced,” relating to the images, feelings, desires, thoughts, and the specific meanings they assume for an individual, and then “life as told,” which is a narrative that is inevitably influenced by the socio-cultural conventions of story-telling.

Idoiaga Mondragon et al. (2022) used drawing as a tool to explore how the COVID-19 lockdown affected children in Spain. They contacted children through their schools’ administration, which forwarded a questionnaire to their parents. Children drew their lockdown experience by answering two questions (“What are you doing during the lockdown?” and “What do you miss?”). Physical activities carried out in the home, daily and routine activities, and art projects were the activities most represented during the lockdown. Most children drew their family members and reported positive emotions, and they reported missing playing and team sports.

## The present study

The present study utilized a retrospective-descriptive design to analyze how children remembered their own lockdown experiences, related their emotions, and how they depicted these through their drawings. This observational work expands on recent drawing-based children COVID-19 research using qualitative and quantitative content analyses and statistical exploration of the association of different responses with gender and school level group.

We aimed to identify, observe, and measure different variables connected to the COVID-19 lockdown experience by addressing the following research questions: how did children represent themselves, other people, places, and objects when thinking back to the times of lockdown [Research Question (RQ)1]? What are the most commonly represented subjects, places, and actions (RQ2)? What emotional content and type of relationships are reproduced (RQ3)? Additionally, in line with the developmental literature, we hypothesized that there would be differences based on gender or school level in the school children’s depictions of their lockdown experiences.

## Materials and methods

### Participants

Sixty classes (72% primary school, 28% middle school) from three school districts in the Umbria region of central Italy took part in the program. This involved 54 teachers who administered it to 906 students [48.8% female, mean age 9.4 years, standard deviation (SD) 1.7 years, age range 7–13 years] after obtaining parental written consent and approval of each school board (Capurso et al., 2021a).

### Measures

The material for this study was drawn from a school re-entry program centered on the possibility for students to share their experiences and emotions connected to the first phase of the Italian lockdown (Capurso et al., 2020, 2021a). The program was built on established crisis management principles for schools, including facilitating classroom discussions and sharing feelings, activities to reconnect socially and with the school’s environment, and sharing of coping strategies that the children used during the lockdown. The program included a course for the teachers and an A4 booklet for children to record, draw and work through their lockdown experiences by writing and drawing their thoughts (see additional materials and appendixes in Capurso et al., 2020, 2021a). When classes carried out the activities, each student completed the seven-page school re-entry booklet to produce a set of personal narratives organized as a continuous storyline, starting at the beginning of the lockdown period and ending in class when school restarted. This resulted in a unique sample of children’s accounts of their experiences.

### Procedure

In September 2020, a group of teachers and their students participated in the re-entry program under the supervision of one of the authors (MC). The teachers took part in a face-to-face four-hour training course. The course explained how to reestablish a sense of school community and outlined the critical role of schools in facilitating the children to process their experiences at cognitive, social, and emotional levels [the training manual is available online in Capurso and Mazzeschi (2020) and is evaluated in Capurso et al. (2021a)]. During the training, the children’s booklet was explained in detail, and instructions were provided for its use in class. The teachers were told to start the program as soon as school reopened and finish it within a few days (the average duration was 5.1 days). One of the booklet’s activities asked children to “Draw a moment that has remained in your mind from when you had to stay at home.” The children could choose any drawing tools they liked to complete the task. The teachers collected the drawings for this paper’s data and they were digitized by the research team.

### Data analysis

We employed a combination of quantitative and qualitative content analysis to analyze the children’s drawings (Huxley, 2020) to respond to RQ1 and RQ2. This technique allows qualitative and inductive determination of constructs of interest and quantitative assessment of their prominence in terms of specific research questions (Krippendorff, 2004). Content analysis can be used on children’s drawings to extract meanings and make sense of the authors’ understanding, attitude, and experience of a specific topic (Milbrath and Trautner, 2008; Wang and Brown, 2019).

We used several scales of the PAIR coding system (Pictorial Assessment of Interpersonal Relationships; Bombi et al., 2007) to address RQ3. PAIR is a psychological instrument developed to organize and code drawings representing relationships and emotions. Each of the instrument’s six scales can be used independently. First, we used the Scale of Value to identify the “self” in the picture, assuming that this was the character with the highest score based on a set of attributes (dimensions, position on the page, body details, and colors). Then, in line with RQ3, we used the following scales:

1. Emotions (centered on the character assumed to represent the self), based on the assessment of graphic clues expressing one of the following nominal and mutually exclusive items; neutrality, contentment, hostility, and discontent.
2. The Emotional Climate (used when two or more subjects were represented), which we reduced to two mutually exclusive nominal categories: sharing the same emotional state or presenting different emotional states.
3. Cohesion, which measures the interdependence between the partners (when more people were present), and provides a score from 0 to 6, based on the presence of six pictorial cues (looking, approaching, acting together, being near each other, sharing a common location, touching each other, or being connected by an object).
4. Distancing, which measures the autonomy of the partners, and provides a score from 0 to 6 based on the presence of six pictorial cues (avoiding looking at the other person, moving away, acting independently from the other person, being far, staying in a specific space (not shared with the other person), and being separated by something).
5. Conflict, which informs on the disruption of the relationship.

Pictorial assessment of interpersonal relationships is based on marking qualitative characteristics in a picture and its represented subjects from a set of described pictorial features. PAIR is a viable tool to study children’s representations of their social world, and its development has followed rigorous validation (Bombi et al., 2007).

#### Development of a coding scheme for the contents analysis

The first and last authors qualitatively classified the analytical constructs by inductively assessing the participants’ drawings to develop the coding scheme. The scheme reflected basic drawing characteristics (e.g., color and framing of the subjects) and what the authors believed to be critical content in relation to the lockdown experience (e.g., loneliness, screentime, and social activities), based on the pandemic situation and current literature on the subject. The complete coding scheme is shown in Table 1. Next, the authors generated a codebook for the analysis of the drawings (see Supplementary File 1). Different codes were created concerning the distinctive themes that were identified in the initial coding scheme (e.g., the theme “what is represented” was divided into the following codes: only the main subject, the main subject with other people, only objects, pets, COVID/death). Subsequently, transcripts from 40 randomly selected drawings were open−coded by the first author and another research team member to generate preliminary codes. Interrater reliability analysis was performed on this subsample by calculating Cohen’s kappa (κ) for each code, resulting in consistency values between 0.72 and 0.94 across the codes. The coders discussed coding differences and identified and described any characteristics or details they believed carried the same meaning. The codebook comprised the code with a short descriptive label, a definition of the concept, a list of criteria for inclusion or exclusion, and examples from the children’s drawings. In this phase, atypical or uncommon answers (<5%) were either grouped with other similar codes or were added to an “other” category. Once the initial codebooks had been generated, the answers were coded independently, and the authors met regularly to refine the definitions and codes. Summaries of the final codes, definitions, and exemplary drawings are presented in the attached codebook as Supplementary File 1. The original dataset (in Italian and Filemaker format) is available upon request from the corresponding author.

**TABLE 1:** Overview of the coding scheme developed for the drawings.

Theme	Code
Color	Color
	Black and white
Framing of the main subject	Full body or face
	No main subject (sbj); sbj with a back view; sbj on screen
Represented place	Inside the house
	Surroundings of the house
	Other; outdoor or cityscape
External world	Not visible
	Partially visible
	Most/all of the scene is external
Activity of the main subject	Play, sport
	Use of ICT
	Daily chores, daily house activity
	ICT/online school
	Escaping the present
	Other
What is represented?	Only the main subject (self)
	Main subject and other people
	Only objects, no people
	Pets
	ICT
	COVID, death
How is ICT represented?	ICT with no people
	ICT with distance school
	Active use of ICT
	Passive use of ICT

Sbj., subject; ICT, information and communication technology. See the codebook (additional material) for a full definition and a detailed description of each code.

#### Statistical analysis

Most of the codes were treated as nominal variables and were described in terms of the overall frequency of occurrences and percentage; additionally, the number of occurrences and relative percentage were also reported separately based on school level [primary school (PS) vs. secondary school (SS)] and gender (males vs. females). A Chi-square test (χ^2^) was performed to assess differences in code occurrences between gender and school-level groups. Given that the two PAIR scales of Cohesion and Distancing produced an ordinal score, a paired *t*-test was run to analyze the differences between Cohesion and Distancing in the total sample, while the differences in the Cohesion and Distancing scores between school level and gender for each one of the scales was calculated using the Univariate Analysis of Variance (ANOVA) with two fixed factors. All analyses were carried out with Statistical Package for Social Science (IBM Corp, 2019).

#### Qualitative content analysis of the drawings

The quantitative content analysis allowed us to determine the statistical relevance of specific drawing details; however, the depth, richness, and texture of children’s drawn experiences were lost within the broader codes used by the quantitative content analysis. Therefore, we conducted a secondary, in-depth qualitative inductive content analysis (Miles and Huberman, 1994) on selected drawings representing each of the analyzed codes or a relative overarching theme. For the qualitative analysis, the authors selected all the pictures that corresponded to a specific code, displayed them in a gallery, and jointly discussed and commented on them to select what they felt was most meaningful. The units in this analysis were the things appearing in the drawings, their shapes and colors, the people, their expressions, and their activities. The researchers were more interpretive in this stage; they formed questions and wrote conceptual comments on how the chosen picture, the COVID-19 pandemic, and the lockdown context would be connected in the representation of the child’s reality.

## Results

Of the 906 participants, 900 completed the drawing activity. The drawings were analyzed based on the coding scheme (Table 1), and the main results are reported in Table 2. As the qualitative content analysis of the drawings inevitably deals with the same principles, relationships, and generalizations as the quantitative data, our comments from the qualitative analysis have been integrated into the following results paragraphs.

**TABLE 2:** Quantitative content analysis of the drawings for school level and gender.

*Code*	*N. 900 Total (%)*	*PS (n* = *583)* *(%)*	*SS (n* = *317)* *(%)*	*χ 2*	*P-value*	*Male* *(n* = *437)* *(%)*	*Female (n* = *463)* *(%)*	*χ 2*	*P-value*
Color	679 (75.44)	501 (85.93)	178 (56.15)	98.32	**<0.001**	327(74.83)	352 (76.03)	0.17	0.676
Black and white	221 (24.56)	82 (14.07)	139 (43.85)			110 (25.17)	111 (23.97)		
**Framing of the main subject**									
Full body or face visible	643 (71.44)	463 (79.42)	180 (56.78)	47.42	**<0.001**	313(71.62)	330 (71.27)	0.014	0.907
No subject, no face, on-screen, other	257 (28.56)	120 (20.58)	137 (43.22)			124 (28.38)	133 (28.73)		
**Represented place**									
Inside the house	613 (68.11)	385 (66.03)	228 (71.92)	2.75	0.097	286 (65.45)	327 (70.63)	2.77	0.095
Surroundings of the house	151 (16.77)	114 (19.55)	36 (11.35)	10.30	<0.001	83 (18.99)	69 (14.90)	2.40	0.121
Other, outdoor or city	136 (15.12)	84 (14.41)	53 (16.72)	0.64	0.424	68 (15.56)	67 (14.47)	0.209	0.647
**External world visible?**									
Not visible	532 (59.11)	325 (55.75)	207 (65.30)	7.75	**<0.05**	245(56.06)	287 (61.99)	3.26	0.071
Partially visible	110 (12.22)	73 (12.52)	37 (11.67)	0.14	0.710	57 (13.04)	53 (11.45)	0.534	0.465
Most/all of the scene is external	258 (28.67)	185 (31.73)	73 (23.03)	7.61	**<0.05**	135(30.90)	123 (26.56)	2.058	0.151
**Activity of the main subject**									
Play, sport	250 (27.77)	200 (34.31)	50 (15.77)	35.15	**<0.001**	123(28.15)	127 (27.43)	0.05	0.810
Personal use of ICT**	238 (26.44)	147 (25.21)	91 (28.71)	1.38	0.238	115 (26.32)	123 (26.57)	0.00	0.935
Daily chores or routines	134 (14.88)	99 (16.98)	35 (11.04)	5.71	**<0.05**	62 (14.19)	72 (15.55)	0.33	0.565
Distance learning***	72 (8)	32 (5.49)	40 (12.62)	14.18	**<0.001**	28 (6.41)	44 (9.50)	2.92	0.087
Escaping the present	54 (6)	35 (6)	19 (5.99)	0.00	0.995	25 (5.72)	29 (6.26)	0.11	0.731
Other or not applicable	152 (16.89)	70 (12.01)	82 (25.87)	nc	nc	84 (19.22)	68 (14.69)	nc	nc
What is represented?*									
Only the main subject (self)	482 (53.55)	317 (54.37)	165 (52.05)	0.44	0.504	239 (54.69)	243 (52.48)	0.44	0.507
Main subject and other people	266 (29.55)	197 (33.79)	69 (21.77)	14.26	**<0.001**	120(27.46)	146 (31.53)	1.79	0.180
Only objects, no people	145 (16.11)	66 (11.32)	79 (24.92)	28.10	**<0.001**	76 (17.39)	69 (14,90)	1.030	0.310
Pets	103 (11.44)	82 (14.07)	21 (6.62)	11.21	**<0.001**	39 (8.92)	64 (13.82)	5.32	**<0.05**
COVID, death	66 (7.33)	37 (6.35)	29 (9.15)	2.37	0.123	27 (6.18)	39 (8.42)	1.66	0.196
Other	8 (0.88)	2 (0.34)	6 (1.89)	nc	nc	4 (0.92)	4 (0.86)	nc	nc
**How is ICT represented?**									
Passive use of ICT	155 (17.22)	97 (16.64)	58 (18.30)	0.40	0.529	87 (19.91)	68 (14.69)	4.3	**<0.05**
Only ICT, no people	86 (9.56)	35 (6.00)	51 (16.09)	24.16	**<0.001**	45 (10.30)	41 (8.86)	0.541	0.462
Active use of ICT	83 (9.22)	50 (8.58)	33 (10.41)	0.82	0.364	28 (6.41)	55 (11.88)	8.04	**<0.05**
ICT as part of distance learning	72 (8)	32 (5.49)	40 (12.62)	14.18	**<0.001**	28 (6.41)	44 (9.50)	2.93	0.087
***Total of drawings containing ICT***	396 (44)	214 (36.71)	182 (57.41)	35.73	**<0.001**	188(43.20)	208 (44.92)	0.33	0.565

*The codes reported under this theme section are not mutually exclusive. **This code encompasses active or passive use of ICT, as reported separately under the ICT representation theme.

***This code is also reported as part of the ICT representation theme and has been repeated here for information completion of the depicted activities. PS, primary school; SS, Italian first degree of secondary school; ICT, Information and Communication Technologies; nc, not calculated. Bold font indicates statistical significance.

### Color, subject framing, and representation

**FIGURE 1:**
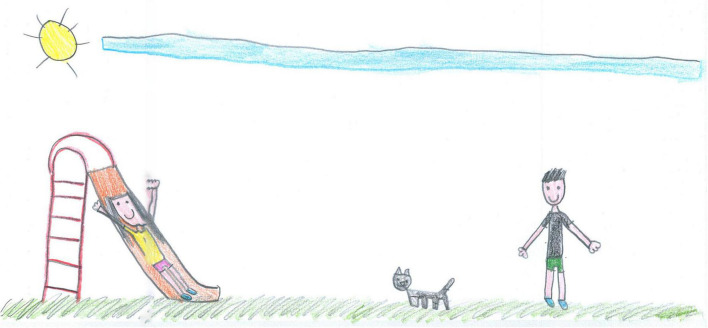
A colorful representation with full body view and other people present (PS, Female, 7 years).

**FIGURE 2:**
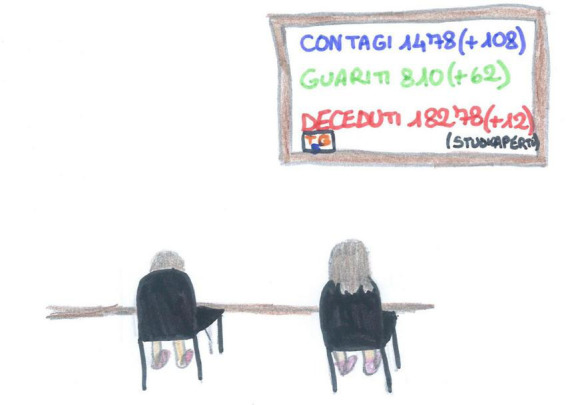
An example of back view representation. The screen is reporting on the increase of the contagions, recovered people and death (SS, Female, 11 years).

**FIGURE 3:**
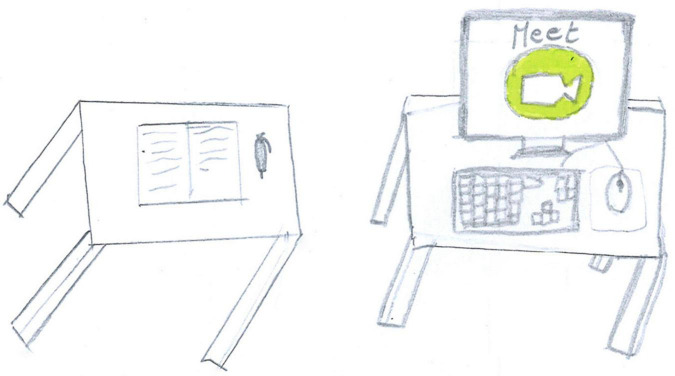
Another different example of a drawing with no people. A large white space is filled only with a computer displaying a videocall program and a table with a notebook and a pen, but there is no-one there to use them (PS, Female, 10 years).

**FIGURE 4:**
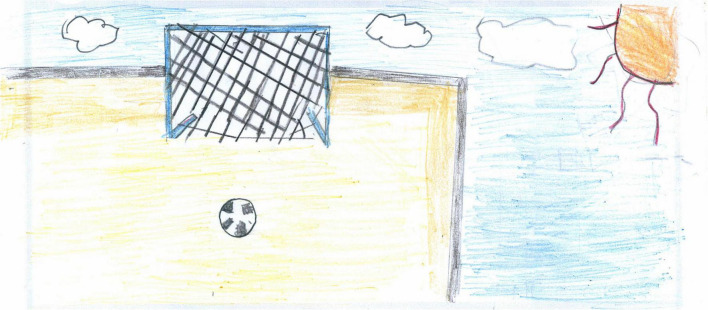
A different example of a drawing with no people. The playfield is empty, but the drawing is full of color, sunlight, and that ball in front of the net is “asking” to be kicked soon (PS, Male, 10 years).

**FIGURE 5:**
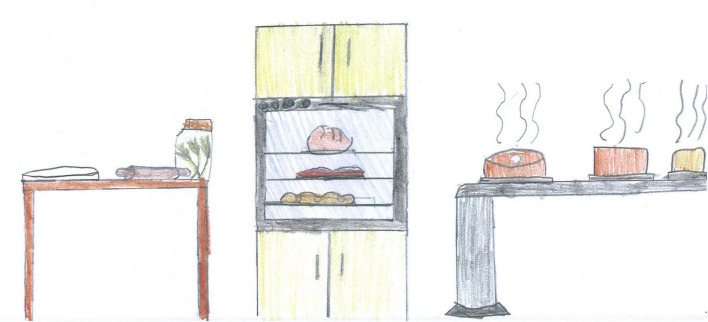
An example of missing people; this time the depicted scene is the inside of the house. Someone must have prepared those cakes and surely is going to eat them soon (PS, Female, 10 years).

Most children used colorful representations (75%) with a full-body view (64%). There was a higher prevalence among primary school (PS) students [color: χ2 (1) = 98.32, *p* < 0.001; full-body: χ2 (1) = 48.45, *p* < 0.001; see Figure 1]. In 42% of the pictures made by SS students, the self was either missing or represented by a back view, χ2 (1) = 42.27, *p* < 0.001 (see Figures 2–5). Other people were also present in 61% of the drawings.

**FIGURE 6:**
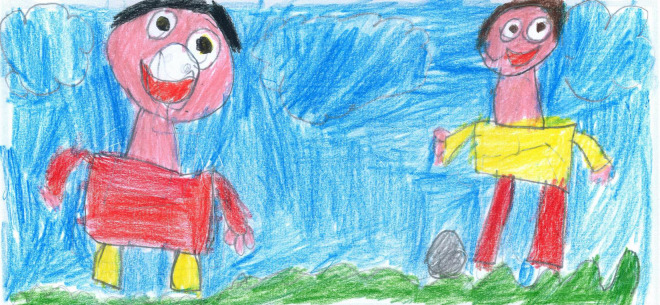
When a play buddy is present, the illustrations are richer in color and details. According to the PAIR system, this picture also has a high cohesion ranking because the children stand on a common area, share the same activity, and are moving toward each other (PS, Male, 8 years).

**FIGURE 7:**
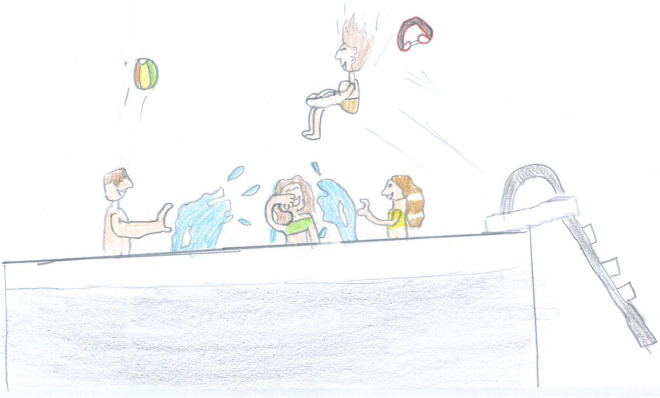
A lucky boy who could access a backyard swimming pool (SS, Male, 11 years).

**FIGURE 8:**
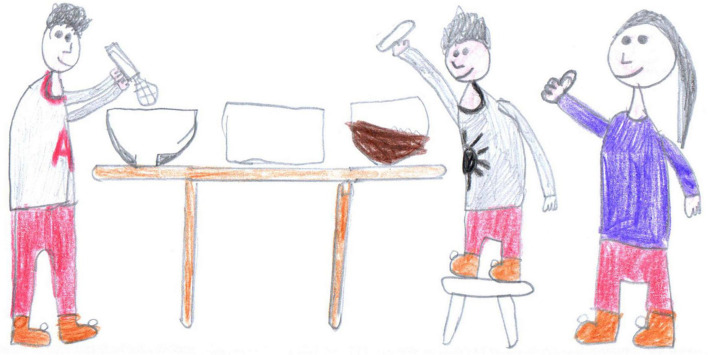
Domestic chores, two children helping in the kitchen (SS, Male, 11 years).

**FIGURE 9:**
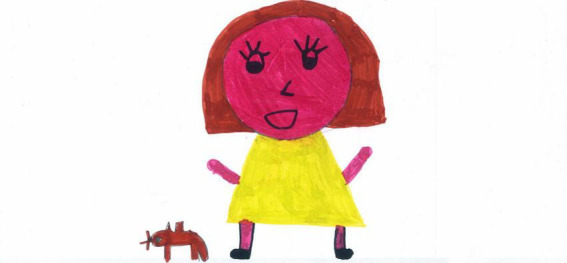
A young girl is playing with a pet (PS, female, 7 Years).

**FIGURE 10:**
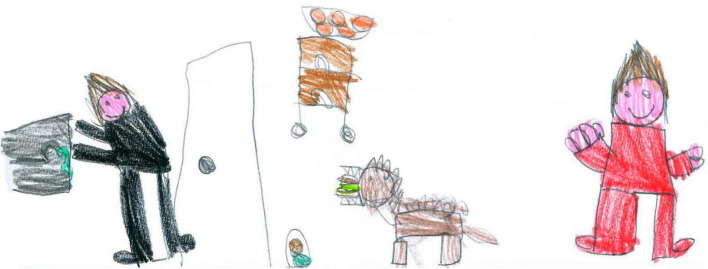
A picture with a high score of distancing according to the PAIR scale. The subjects are separated and far apart, are doing a different activity, and are moving away from each other (PS, Male, 7 Years).

For example, colorful and well-detailed images are present in Figures 1, 6–10. A large part of the drawing area has been used in these images. These pictures convey a sense of completeness and satisfaction; they show a world filled with friends, pets, and play activities that allowed the child to navigate through the hard times, despite the isolation of the lockdown. The images communicated by these drawings may not necessarily reproduce a lived reality but reflect an internal world capable of remaining active and well organized despite the crisis situation.

Figures 2, 3 show different types of void representation where people (or faces) are invisible. Only technology is present, but even where two people are represented (Figure 2), they are static. They disappear on the sofa, and the space is overwhelmed by the invasive presence of the television (TV) (which is showing deaths and COVID-19 case counts). In both pictures, an empty, white space dominates.

The absence of people can also convey a positive expectation, like in Figures 4, 5. For a 10-year-old Italian boy, the affordance of that ball in front of the net in Figure 4 on a football field is irresistible. That ball is “asking” to be kicked and will be as soon as the lockdown is over, and the field will be filled with boys and girls running and shouting, rejoicing at their regained freedom. The boy is probably projecting all his desire to go out and play with his friends again, and his mind is reassured by the representation of a world that is still there and waiting for his return. In Figure 5, someone has just finished baking those hot pies and will be eating them shortly. Both these pictures depict elements of life even in the absence of human figures; they are full of color and warmth and convey positive expectations and a sense of security or community.

### Represented activities

#### Screentime and information and communication technology use

**FIGURE 11:**
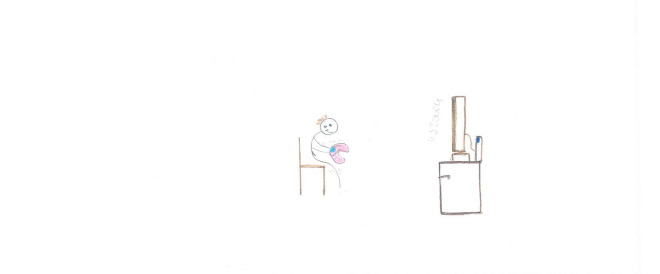
A child is passively playing videogames. The body is crushed, folded back on itself and chained to his seat (PS, Male, 10 Years).

**FIGURE 12:**
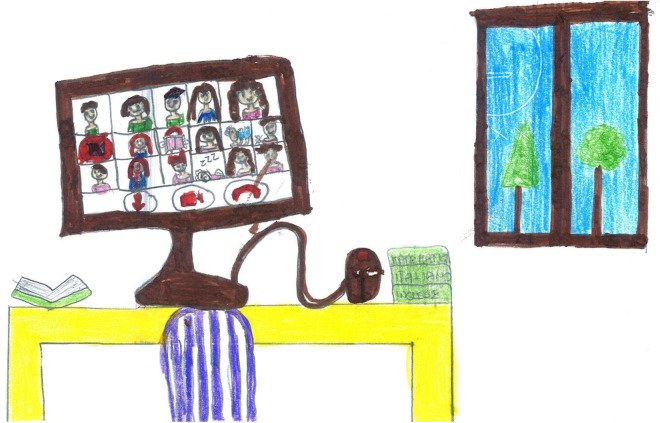
Distant learning, but the girls’ own chair is empty (SS, Female, 11 years).

**FIGURE 13:**
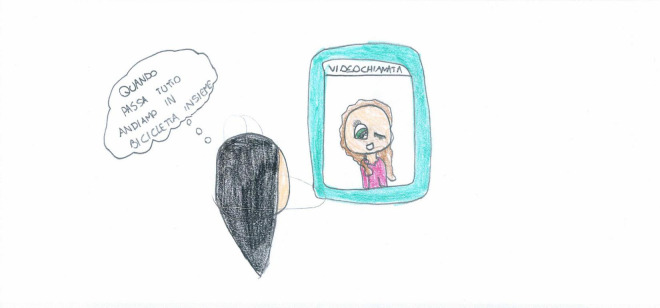
An example of two girls actively using ICT for a chat. The girl is saying: “when this is over, we’ll ride together in our bicycle” (PS, Female, 10 years).

Screentime activities (44%) were the most represented (Figure 2), with an overall prevalence among SS children [χ^2^ (1) = 35.15, *p* < 0.001]. Given their high usage during the lockdown, we conducted an in-depth analysis of the diverse screentime representations. Technology was used passively by most children (17.22%, Figure 11), and information and communication technology (ICT) was depicted without any people present in another 9.56% [this was mostly SS, χ^2^(1) = 24.16, *p* < 0.001, Figure 3]. Older children also represented distance learning more often [χ^2^(1) = 14.18, *p* < 0.001, Figure 12]. Finally, 9.22% of the children also used technology more actively (e.g., chatting and working on the computer). Figure 13 shows an example of the use of technology as a means of positive communication; two girls are using a chat app to stay in touch with each other and make plans. The face of the friend is colored and full of details. The subject’s face is not visible, but her hair appears brushed and well-kempt. The white space around gives us a sense of isolation, but the relationship with a friend, placed in the middle of the sheet, appears to alleviate this.

An ambivalent function of technology is visible in several drawings. For example, Figure 12 shows a distant learning activity, with a desk ready and books and images of school friends on the screen. However, the main subject, the author of the drawing, is absent, and her chair is empty. Another example of emptiness can be seen in Figure 11, where a child plays a video game. This time the self is present, but the body is just an outline, bent over, alone, ensconced in front of the screen. Figure 3 carries an additional paradox because it depicts a screen with a renowned communication app. The problem is that no one is present to use it. In this case, the emptiness of the room is reflected in the emptiness of cyberspace.

#### Other activities

**FIGURE 14:**
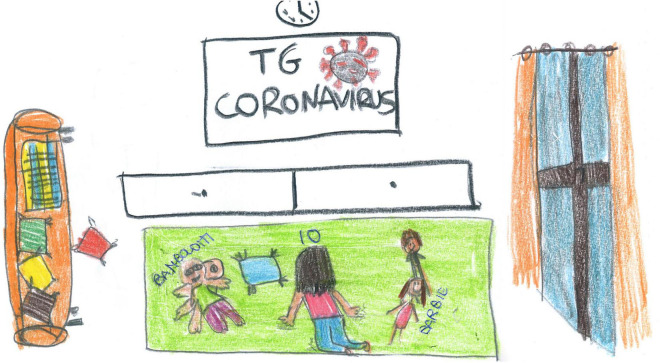
Playing at home. The TV screen is showing the pandemic news, but the children are playing inside a separate area, marked by the green carpet, that seems to keep them safe from the menacing world (PS, Female, 10 years).

**FIGURE 15:**
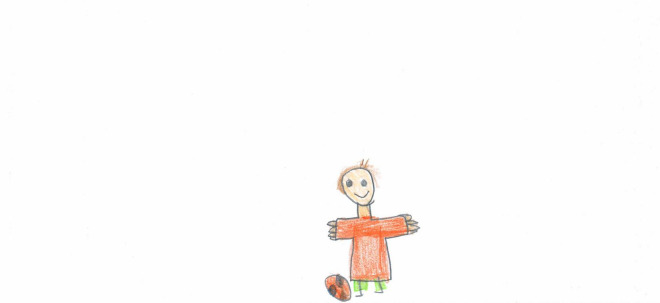
Playing with a ball. A lonely child is playing with a ball, amid a large white empty area. Yet, he is smiling (PS, Male, 7 years).

**FIGURE 16:**
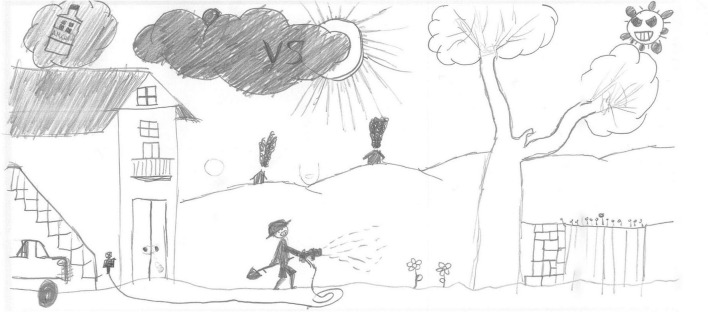
Taking care of the garden (SS, Male, 11 years).

After screentime, PS students depicted play, sport or physical activities (28%, Figures 6, 7, 14, 15) [χ^2^(1) = 35.15, *p* < 0.001]. Figure 14 shows a young girl playing alone. The carpet clearly demarks the play area, which draws a clear line between a play world and a more menacing outside world, represented by the TV screen reporting on the COVID-19 pandemic. Even if the girl’s face is not visible, all the dolls are smiling. Next to the carpet is a shelf with more play boxes (one of them has been chosen and taken to the middle of the carpet), showing that a large reserve of play activities is available. This young girl shows how play was important to her and how it generated a safe area represented by the carpet, where smiles were still present. They probably represent a projection of a good inner part of the girl’s self that she can access and use as a comfort in difficult times. In other pictures where children are playing together, they are always smiling (Figures 6, 7, 10). Domestic routines (15%) were also reported; Figure 8 shows two children preparing a recipe on a kitchen table. They are probably siblings and are working under their mother’s vigilant (yet benevolent) eye. These children are taking part in what Bronfenbrenner (1979) would call a dyad based on a joint activity, a relationship that is mediated by the cooperative task of making the recipe. All the family members are smiling, showing shared positive emotions, and wearing trousers of the same color. The outside world is not visible, but life in this family looks harmonious despite the father’s absence. Figure 16 is more ambiguous. It shows a boy watering a lawn in front of his house. The picture is in black and white and conveys all the insecurity connected to the first stage of the COVID-19 pandemic. The sun is partially obscured by the clouds, and at the top right side of the picture, a grinning and intimidating virus is watching. But the picture also contains elements of life. Two flowers are thriving, and the houses in the background emit smoke from their chimneys to reflect the life that is inside. Hovering over the boy’s house is a bottle of disinfectant that keeps the virus away.

**FIGURE 17:**
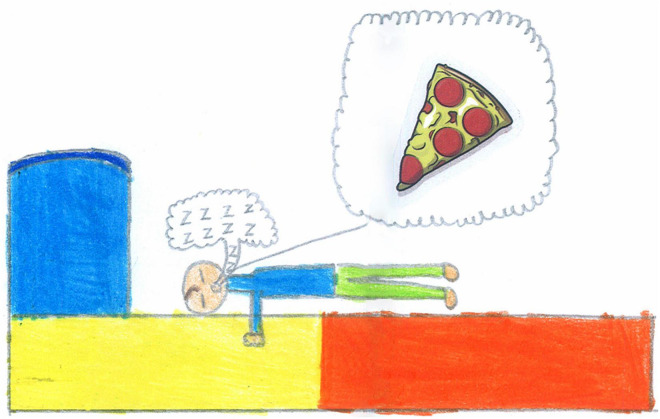
Dreaming of a pizza… (SS, Male, 11 years).

Finally, 6% of children represented escaping activities such as dreaming; Figure 17 shows a boy dreaming of a slice of pizza, a typical longed-for snack among Italian children during the lockdown.

### Places

Most children (68.11%) drew the interior of their houses (Figures 2, 3, 5, 11, 12, 14, 17). The outside world was completely absent in 59.11% of the pictures, with a slight prevalence among SS [χ^2^ (1) = 7.75, *p* < 0.05]; For example, in Figures 5, 8, 11, the absence of the external world amplifies the sense of intimacy and isolation of the subjects. Sometimes (10.44%), the external world was visible through a window or part of the picture, as in Figure 12. This reminds the child of the life outside that continues despite the lockdown and is waiting to welcome the subject once the isolation is over.

In terms of depicted objects and living creatures, the self was represented alone in 53.55% of the images (Figure 15); when it was associated with other people (29.55%), this was more often done by PS children [χ^2^ (1) = 14.26, *p* < 0.001; see Figures 2, 6). Pets (11.44%, Figure 9) were more often portrayed by PS [χ^2^ (1) = 11.21, *p* < 0.001] and females [χ^2^(1) = 5.32, *p* < 0.05].

Figure 9 shows a young girl playing with her pet. The girl’s body and head dominate compared to the rest of the picture, colors are marked and joyful, and the surfaces are filled with color to confirm the presence of a solid self. The child’s arms are elevated to show an overall sense of agency. Even if the rest of the world is not visible, the presence of the pet holding a stick reminds the girl of her time playing fetch with her pet. Not everyone had an open space and a pet to play with. Figure 15 shows a child playing alone with a ball. White emptiness is prevalent in this picture. The subject occupies a small portion in the middle, and he is lonely. Despite this, he is smiling, his arms are open as if to embrace the world, and his body is filled with color and details. This picture is a testimony to the resilience that younger children could show, even when they had no siblings or friends to play with during the lockdown.

### Emotion and relationships analysis with the pictorial assessment of interpersonal relationships scale

The PAIR scale could be applied only to 257 drawings that depicted two or more people; therefore, the following data only apply to this subsample (55% female, mean age 9 years, SD 1.6 years, age range 7–13 years).

#### Emotions

**FIGURE 18:**
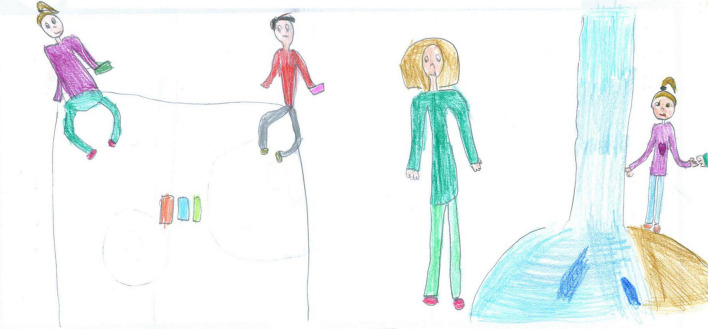
PAIR’s shared neutrality (PS, Female, 10 years).

**FIGURE 19:**
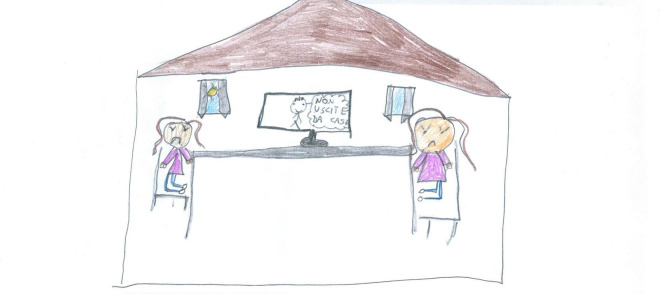
An uncommon example of PAIR’s negative emotions; TV says: do not go outside (PS, Female, 9 years).

Contentment (i.e., positive emotions) was expressed in most of the PAIR-analyzed drawings (60.93%, see Figures 1, 6–9, 14, 15), with a prevalence among PS [χ^2^(1) = 11.88, *p* < 0.001] and females [χ^2^(1) = 5.95, *p* < 0.05]. Neutrality (33.98%, Figure 18) was more common among SS [χ^2^(1) = 9.43, *p* < 0.05] and males [χ^2^(1) = 8.93, *p* < 0.05]. Discontent (Figure 19) or hostility was seldom detected (5.09%).

Examples of shared positive emotions can be seen in Figures 1, 6–8, 10. These pictures show smiling children playing or engaged in some other collaborative activity. The colors are well distributed and bright. The people have a positive attitude, and the whole image paints an optimistic picture of the represented event.

Conversely, Figure 19 depicts a rare example of shared negative emotions; two young girls sit at a table with a TV between them, reminding them they cannot go out. The color fill is less uniform than in other pictures, and the TV and long table increase the distance between the girls, who appear unable to play together and are left alone with their anger and sadness. The external world is still present since a blue sky and a shining sun are visible through the window, but these act as reminders of what the girls are missing and wishing for.

#### Emotional climate

The represented subjects often shared the same emotion (59.14%, see Figures 1, 6–8, 14), with a prevalence among PS [χ2(1) = 13.69, *p* < 0.001] and females [χ2(1) = 10.55, *p* < 0.001].

Figure 1 is an example of sharing positive emotions. It depicts a couple of children playing outdoors with a pet. Albeit distant, the children are smiling, and their whole body is visible in color with full details; the sky is blue, the sun is shining, the grassed ground fills the bottom of the page, and the drawing space is filled with details and colors. Despite some empty background, this picture was manifestly conceived by a young girl who experienced positive times during the lockdown and could most likely access open space to play with friends or siblings.

**FIGURE 20:**
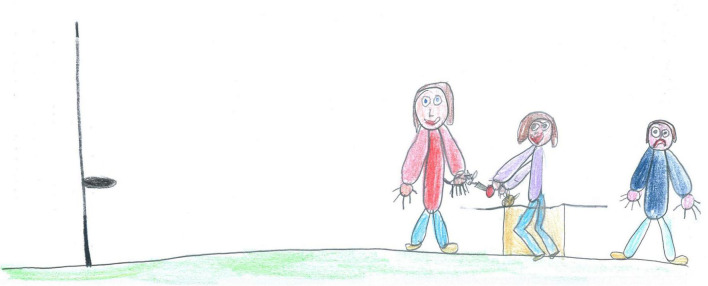
An example of PAIR’s opposite emotions (PS, Male, 8 years).

Shared neutrality (30.74%, Figure 18) was more frequent among SS [χ2(1) = 6.56, *p* < 0.05] and males [χ2(1) = 8.67, *p* < 0.05]. Unilateral or opposite emotions were rare (10.12%; Figure 20 is an example of opposite emotions). The child on the right is somehow excluded from playing with the other two subjects in the middle of the drawing. The child’s body is blue, and he shows a sad expression. This picture reminds us that not all children live in a happy family environment without tensions and conflicts, and for some children, the memory of the lockdown is connected to difficult or negative experiences.

#### Conflict

Virtually no conflict (97.67%) was depicted in the pictures analyzed.

#### Cohesion and distancing

The descriptive and ANOVA results for these scales are reported in Tables 3, 4. A *t*-test (*t* = 2.62; *p* < 0.01) showed that children reported significant higher cohesion (mean 2.15 ± 1.18) than distancing (1.82 ± 1.48). Moreover, the results revealed no significant differences between males and females (*p* > 0.05) or between PS and SS students (*p* > 0.05). For example, Figure 6 is an image with a high cohesion score because children are participating in coordinated play activity, moving toward each other, standing in a common area of the playfield, and are looking toward each other. On the other hand, Figure 20 shows a picture with high distancing, where the child in blue is separated from the other two subjects who are playing together and appear to be walking away and not looking at him.

**TABLE 3:** Quantitative content analysis of the selected PAIR items for emotions, relationships, and conflicts (*N* = 257).

*Code*	*N. Total (%)*	*N. primary* *(%.)*	*N. Secondary (%.)*	*χ 2*	*P-value*	*N. Male* *(%)*	*N. Female* *(%)*	*χ 2*	*P-value*
									
**Emotions**									
Contentment	156 (60.93)	127 (66.84)	29 (43.28)	11.88	**<0.001**	60 (52.17)	96 (67.61)	5.95	**<0.05**
Neutrality	87 (33.98)	54 (28.42)	33 (49.26)	9.43	**0.05**	50 (43.48)	37 (26.06)	8.93	**<0.05**
Discontent or hostility	14 (5.09)	9 (4.74)	5 (7.46)	nc	nc	5 (4.35)	9 (6.33)	nc	nc
**Emotional climate**									
Shared emotions	152 (59.14)	125 (66.14)	27 (40.30)	13.69	**<0.001**	55 (47.83)	97 (68.31)	10.55	**<0.001**
Shared neutrality	79 (30.74)	50 (26.46)	29 (43.28)	6.56	**<0.05**	46 (40.00)	33 (23.24)	8.67	**<0.05**
Unilateral or opposite emotions	26 (10.12)	15 (7.4)	11 (16.42)	3.96	**<0.05**	14 (12.17)	12 (8.45)	0.33	0.97
**Conflict**									
No conflict	251 (97.67)	187 (98.42)	64 (95.22)	1.825	0.177	112 (97.39)	139 (97.89)	0.069	0.793
Some form of conflict	6 (2.33)	3 (1.58)	3 (4.78)	nc	nc	3 (2.61)	3 (2.11)	nc	nc

nc, not calculated. Bold font indicates statistical significance.

**TABLE 4:** Univariate analysis of variance of PAIR cohesion and distancing scales – based on school level and gender (*N* = 257).

	Total	PS	SS			Male	Female		
							
	Mean ± SD	Mean ± SD	Mean ± SD	*F*_(1,257)_	*P-value*	Mean ± SD	Mean ± SD	*F*_(1,257)_	*P-value*
Cohesion	2.15 ± 1.18	2.18 ± 1.23	2.07 ± 1.03	0.215	0.643	2.01 ± 1.16	2.27 ± 1.18	2.49	0.116
Distancing	1.82 ± 1.48	1.89 ± 1.51	1.64 ± 1.40	1.32	0.252	1.80 ± 1.57	1.84 ± 1.41	0.029	0.866

PS, primary school; SS, secondary school.

## Discussion

This study analyzed a set of Umbrian-Italian children’s lockdown-related drawings, which were retrospectively created in September 2020 when the school was restarted after the first COVID-19 wave. The results of this research offer several insights into how children experienced their first lockdown and how they narrated their lives at home through drawings. The combination of quantitative and qualitative content analysis enabled an in-depth analysis of the children’s vision and perceptions of the situation.

The 900 drawings show that most children, despite the difficulties caused by the pandemic, remembered and depicted a happy scene from the lockdown experience. The prevalence of colorful pictures, full-body framing, contentment, play, and family life, convey a positive message. It is important to note that the children in our sample spontaneously drew positive memories of their time at home. The images show that the perceived psychological outcomes of the lockdown are not a causal consequence of the crisis event *per se* but are mediated by systemic factors that affect people and family life (Ford and Lerner, 1992). This finding provides opportunities for support intervention that should and could be organized and sustained by public and community organizations. For example, Singh et al. (2020) recommend activities and interventions involving parents, teachers, pediatricians, community volunteers, the health system, and policymakers. At the children’s level, recommendations include engaging youngsters in play activities, communicating with children in an age-appropriate manner about the pandemic situation, limiting their exposure to unfiltered news, providing stable family routines, and outlining possible activities rather than prohibitions. For adolescents, the recommendations include modeling important life skills like coping and problem-solving, transmitting a sense of control whenever possible, and allowing older children to learn responsibility, accountability, involvement, and collaboration in daily tasks.

The generally positive images of the lockdown found in our study contradict the many pieces of research reporting on fear, anxiety, and depressive problems among children and adolescents due to the COVID-19 pandemic (for a review, see Samji et al., 2022; Viner et al., 2022). One possible reason for this discrepancy can be traced back to the socioeconomic characteristics of our sample. Despite not performing a socioeconomic analysis of the participants due to privacy limitations, the schools in this study were all based in the Umbrian countryside. This area is usually populated by middle- or working-class families, mostly single or double households, with easy access to a backyard or the countryside. Living in a rural area was a protective factor against anxiety, while spending time creatively during the lockdown was significantly protective against experiencing negative emotions and emptiness (Forte et al., 2021).

Another reason why children reported positive memories of the lockdown could be traced back to the metamodel underlying the present research (Ford and Lerner, 1992). Most clinical studies of children’s reaction to the lockdown (Panchal et al., 2021) were based on a bio-medical model of “deviations from the norm → diagnosis” (Engel, 1977). They hypothesized adverse mental health outcomes resulting from the pandemic, purposely looked for them and (consequently) found them (Telfener, 2011). In fact, clinical studies are usually based on close-ended questions and prompts, often “suggesting” a specific list of symptoms for the child to choose from. In contrast, our study is based on a more systemic, Whole Child Development view of human experience (Cantor et al., 2021). This approach postulates that contexts, relationships, and environments (experienced in different life settings) are the primary determinants of human development (Cantor et al., 2021). With proper care from supporting, dependable adults and environments, the adverse developmental effects of crises are preventable and reversible; children can overcome the negative effects of adversity and thrive (Cantor et al., 2021). In line with this approach, we hypothesized that despite the adversities, children would have found ways of thriving and exhibited resilience, coping capabilities, and emotional processing skills. Therefore, the prompt asking children to draw their experience was neutral in the present study to allow the subjects complete freedom to choose what to draw and what type of experience to report (e.g., positive or negative).

As a result of our method, most children chose to depict a cheerful moment. Our quantitative data appear to suggest several co-factors contributing to a positive recall of the lockdown experience. These include play and physical activities, taking part in an activity with other family members, interacting with a pet, and actively using ICT for peer communication. These factors can all be traced to a common crucial developmental denominator; an active, affective relationship with others. Children who depicted contentment were usually engaged in a task that involved being in a relationship with someone. This often happened within the family, when siblings or caregivers were readily available, around the house with friends, when open spaces were accessible, or even in a solitary play world such as the ones depicted in Figures 14, 15.

Another methodological factor that differs from other clinical studies is the choice to give voice directly to the children. Most previous research reporting on negative mental health outcomes of the pandemic was based on proxy reports (Samji et al., 2022); therefore, these studies reported on the *perception of an informant* on what children felt and how they behaved. The literature shows that informant discrepancies are common when evaluating children’s mental health (De Los Reyes et al., 2022), and assessments vary across contexts, and are based on the experience of the person acting as a proxy. An informant would usually respond to questions by reporting on the presence of specific symptoms or problematic behaviors, but this does not reveal what the children actually felt and experienced. Conversely, by giving a voice to children *via* a neutral prompt for them to draw a moment of their own choice from their lockdown experiences, we recognized children’s agency, competence, and the fact that their ongoing interactions with their living and social environment transformed the way they experienced the lockdown (Garbarino and Stott, 1992).

In symbolic terms, there are instances of physical and psychological protection in the children’s drawings in our sample. The most evident physical protection is the representation of the different rooms in the home as a sheltered space where the virus could not enter and where children felt safe and could engage in different activities. Children draw happy and playful moments in the home with one or more family members. This can be seen as a logical consequence of the increased time spent with parents and siblings, but it also indicates the importance of the quality of family life during the lockdown. Not all children were fortunate enough to live in a comfortable house and with a family that was able to buffer them from the risks and stresses of the lockdown (Prime et al., 2020). Risk factors such as financial concerns and uncertainty about the future would have caused some families to be more acutely affected by the socioeconomic consequences of the lockdown (Bérubé et al., 2021).

Another important protective mental space for children was the one opened up by playful activities. In times of stress, play serves several types of behavioral, cognitive, and emotional functions. Play facilitates younger children to assimilate daily experiences and adapt reality to their own thoughts (Capurso and Ragni, 2016). Exploratory play is the basis for learning, achieving goals, and growth. In emotional terms, play has long been seen as a crucial activity to connect with, express, and recognize their own and other people’s emotions (Capurso and Ragni, 2016).

Relationships form the third type of emotional protection. There are three main sources of social support for children and adolescents; family, friends, and school personnel (Chu et al., 2010). The drawings from our sample often depict the presence of family and friends. While the main source of support for younger children is the family, other forms of social support, such as friendships, become more important in older children. Friendships offer several developmental functions, including companionship, stimulation, physical and ego support, social comparison, and intimacy/affection (Ginsberg et al., 1986). Thanks to their interaction with friends, children learn to practice controlling their emotions and responding to the emotions of others (Kouvava et al., 2022), crucial skills during the lockdown.

Generally speaking, younger age and being female are the two independent variables that are most associated with a positive memory of the lockdown times. Remembering a protective experience was more natural for younger children who rely heavily on family life due to their developmental status (Gettler, 2017). Younger children generally drew a relaxed daily life with little constraints in terms of time, space, and content, where enjoyable activities were possible within the safe walls of their homes. For them, the lockdown was often associated with a sense of stability and opportunities to engage in common hobbies, develop greater attachments and enjoy more dialogue with siblings and parents. These findings confirm those of Panchal et al. (2021), who reported that when children were involved in daily routines, had good family relationships, and had access to play, they also felt safe, relaxed, and comfortable within their households. In contrast, peer relationship quality in adolescents tends to become more important than parental relationships (McMahon et al., 2020). They probably lived the lockdown as a time of constraint and limitations to their freedom, and only a few were able to maintain close and intimate contact with their peers. In some cases (Figure 13), ICT helped young people to keep in touch and cultivate their friendships despite the distance. Such active use of ICT was reported more often by girls, who also drew more joyful situations and shared positive emotions than boys. The fact that girls rely more on relationships in times of distress has been well documented in the literature (Belle, 1991). In fact, adolescent girls present higher emotional sensitivity to stressful life events, and consequently, they tend to report higher levels of attachment to peers and favor quality intimate relationships (McMahon et al., 2020).

TV, video games, computers, and smartphones are ubiquitous in the drawings. There is a marked prevalence among SS, and males seem slightly more prone to passive ICT use (Figure 11), whereas females use it more actively (Figure 13). Boredom was a common experience during the lockdown (Melegari et al., 2021), and TV, computer, and video games helped children fill the day. This came with some dangers since the overuse of screentime activities can have harmful consequences, such as sleep disruption, reduced motility, and can lead to an increased risk of psychological difficulties (Lissak, 2018; Nagata et al., 2020). During the lockdown, children with higher levels of screen use had significantly higher levels of mental health symptoms (Li et al., 2021). Our data shows an ambivalent function of the use of technology and the related screentime. On the one hand, technology was sometimes used as a means of positive communication (Figure 13). However, conversely, the large number of images associating ICT with a missing self (Figures 3, 12) seem to show that the intensive use of technology and video games was not perceived as fulfilling the need for friendships and real-life social interactions. Cyberspace was often perceived as a place where the self was dissolved, where faces and emotions disappeared, just like the video camera that was often turned off for privacy and technical reasons during distance learning. This suggests that the code relating to ICT required more in-depth investigations (Table 2). Screentime can be part of an activity that has a purpose, creates relationships, and requires the active involvement of the subject, such as the one depicted in Figure 13. Alternatively, it can be an individual and a closed occupation that creates desolation and, in the end, contributes to the loneliness of the subject (Figures 3, 11). Such ambiguity calls for teachers, educators, and parents to plan the use of screentime and connect it with some activities that promote active relationships. Schools should play a primary role in this kind of planning and should plan, organize, and deliver distance education activities where ICT is used as a tool to reach a final aim or product and not just as a means to deliver traditional subject-based content.

While most drawings convey a positive message, there were instances of negative memories from the lockdown. These were usually connected to drawings showing loneliness/void (Figures 2, 3) or conveying a sense of exclusion and marginalization, as seen in Figures 19, 20.

The pictures with wide empty spaces, indicating a sense of a void, remind us that peer interaction is extremely important to children and that those who lacked it during the lockdown suffered deeply, to the extent that it affected their self-perception. Evidently, not all children had the opportunity for meaningful peer interactions. Children across Europe have reported emptiness as a source of distress (Forte et al., 2021). This data reminds us of the importance of establishing peer support networks in times of crisis and how crucial it is to ensure that such support is available to every child, but especially to those living in families experiencing vulnerability or with a single parent. Such a network could be facilitated by peers, school, or other professional types of interventions.

Support and networking interventions are even more crucial for those children who experienced marginalization, such as those depicted in Figures 19, 20. These images show a household where children did not feel comfortable and expressed their discomfort through pictures showing negative emotions, discontent, low proximity, and high distancing. Supporting families living in vulnerable circumstances is particularly difficult, but evidence-based parenting support programs can be particularly efficacious, especially if they are culturally tailored (Baumann et al., 2015). These programs aim to provide rightful access to high-quality, supportive services that meet the individual needs of each child and family while recognizing the autonomy of each household and respecting each families’ priorities. Strengthening pathways to resilience by providing help for parents and professionals working with children is crucial to consolidating the child and family’s well-being (Fong and Iarocci, 2020). Support programs can intervene through a public agency or community assistance at financial, emotional, and mentoring levels. During a lockdown, they can employ various online, telephone, or physically distanced delivery options to accommodate family schedules and comply with the pandemic restrictions (Perks and Cluver, 2020). Examples of parenting interventions can be found in the Triple P Parent Program (Sanders et al., 2000), which delivered a set of multilevel family support strategies aimed at creating positive relationships between the child and the parents. The program also supports the abilities and development of the child and improves parental skills to manage problematic behaviors. A second widespread intervention is the Incredible Years Parent Program (Webster-Stratton, 1982). This is a comprehensive, multifaceted, development-based intervention that targets parents, children, and teachers to support social skills and prevent or moderate conduct or oppositional defiant disorders, attention-deficit hyperactivity disorders, and emotional or behavioral problems in neglected or abused children.

## Limitations

This study’s main limitations are the limited provenance of the subjects and the inevitably subjective characteristics of the interpretation of the drawings. Due to COVID restrictions, the administration of the task in the classroom could not be controlled by the researcher. We used standardized instructions for the children and their teachers to improve control. We ensured that all the children were administered the task in the same place (their classroom), at a comparable time, and under the same circumstances. To establish confirmability, we used the participants’ drawings to substantiate the interpretations of the data from the quantitative analysis.

Another limitation is connected to the phenomenological nature and timing of this study. Did the children actually *experience* what they had drawn, or did they project fantasies, wishes, and expectations in their pictures? The study’s retrospective nature represents a further limitation since children may have been influenced in their drawing by subsequent experiences between the first wave of COVID-19 and when the data was collected. Indeed, we do not know where the difference between reality and imagination lies in their created images. In some areas of psychology, this difference is of secondary importance. As Thomas and Thomas (1928) stated, if someone perceives a situation as real, its consequences *will be* real. Either way, the drawings and data from this study should not be considered a mirror of reality but rather a personal account of a perceived experience. Moreover, while this study reported on drawings collected from different schools, the sample remains limited to the Umbrian region of Italy. As such, the results are not generalizable to all Italian children. Besides, the COVID-19 experience in Umbria likely differed from other regions, where the first wave of COVID-19 was more dramatic in terms of infections and deaths. Finally, given that culture influences perception and sense-making (Cole, 1998), our findings cannot be generalized to different cultural or geographical settings.

## Conclusion

This study used a child-centered drawings-based approach and prioritized their perspectives and experiences, enabling them to share information in ways that worked for them. This method is considered easily accessed by children of different ages, sex, socioeconomic status, ethnicity, and with different language abilities (Milbrath and Trautner, 2008). In this research, the children’s drawings enabled us to understand how they perceived play, family life, and emptiness during the first wave of the COVID-19 pandemic.

Our results show the protective role of good relationships, play, and happy and relaxed family environments. Technology is depicted in an ambivalent way; sometimes, it contributes to maintaining good relationships, but often it is associated with a dissolved self-image. The connection between ICT-related social functions and the self-image of children and adolescents needs further investigation in the future.

For most of the children in our sample, the lockdown drawings showed positive moments. However, some of the pictures showed signs of discomfort or even distress, usually connected to a lack of peer or family relationships or the absence of the subject’s involvement in meaningful activities. Maintaining developmental trajectories and supporting children’s thriving can be achieved even in times of crisis if family, school, and community support systems are networked and if public policies provide family support and pay attention to those who are more vulnerable.

In conclusion, despite many studies reporting children as having suffered due to the COVID-19 lockdown, our results suggest a different narrative. When presented with the opportunity to recall their lockdown time freely, most children, especially the younger ones, depicted joyful moments, and their pictures transmit a sense of resilience, security, and agency. This could indicate that the difficult times during the lockdown were a natural part of their daily lives. Once everything went back to normal, the children were ready to populate all voids they had drawn during the pandemic.

At this very moment, the young boy who imagined that lonely football in Figure 6 is probably running around and chasing that same ball on a field full of voices, smiles, laughter, shouting, and play.

## Data availability statement

The raw data supporting the conclusions of this article will be made available by the authors, without undue reservation.

## Ethics statement

Ethical review and approval was not required for the study on human participants in accordance with the local legislation and institutional requirements. Written informed consent to participate in this study was provided by the participants or their legal guardian/next of kin.

## Author contributions

MC conceptualized the project, coordinated the data collection, performed the qualitative analysis, and developed the first draft of the manuscript. LB performed the ANOVA and other quantitative analyses, while CM contributed to conceptualizing the work, and supervised the methodology. All authors were involved in writing the manuscript and contributed to the content analysis process.
