# Supplementary material for: Primary and middle-school children’s drawings of the lockdown in Italy
Source: Front Psychol. 2022 Oct 18;13:982654. doi: 10.3389/fpsyg.2022.982654 (PMC9622792; doi:10.3389/fpsyg.2022.982654)
Supplement: Supplementary file 1 [file Data_Sheet_1.pdf]

Codebook for *Children's Drawings of the Lockdown: Narratives of strengths and weaknesses experienced*",

DOI: 10.3389/fpsyg.2022.982654.

	Code	Description	Inclusion and exclusion criteria	Sample pictures
Colorization	Color	At least part of the drawing contains more than one color	Inclusions (I.) Use of more than one color in the drawing<br>Exclusions (E.)<br>The entire drawing has been created using a single color (i.e., pencil, pen etc.).	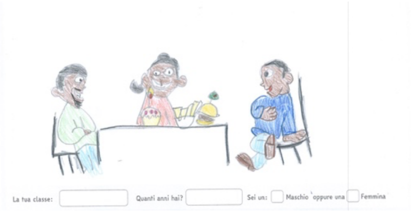
	Black and white	The entire drawing is created using a single color	I. The whole drawing has been done using a single color (i.e., pencil, pen etc.).<br>E. More than one color is visible in at list part of the drawing.	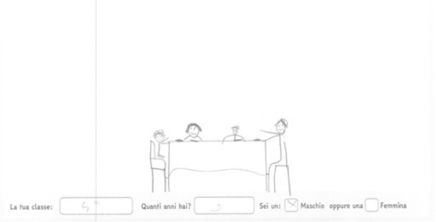
	Identification of the main subject	The following clues are used to identify the main subject:<br>- The only child in the picture<br>- The child in the middle of the picture<br>- The only child of the same sex or gender as reported on the booklet demographic profile<br>- There is a clear indication of the subject identity (i.e., "me")	I. any drawing where a person is represented.<br>E. The drawing does not represent any person.	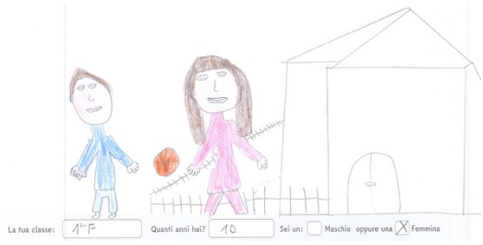 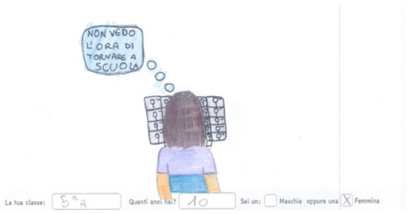

Framing of the main subject	Full body or face visible	The full body and face, or the face alone are visible in the picture	I. Face or full body are visible<br>E. Only body parts are visible with no face; the drawing does not represent any person.	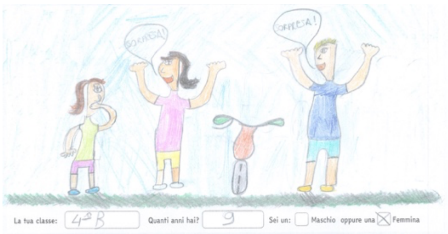
	No sbj, no face, on screen, other	The back of the person or only some body parts are visible	I. Back of the head, shoulders, or some body parts of the person are visible. The drawing does not represent any one person.<br>E. Full body or face is visible.	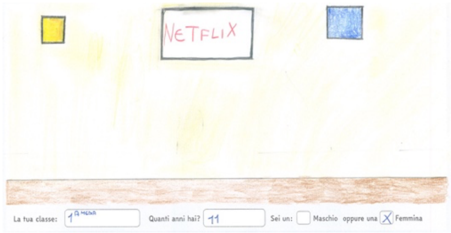 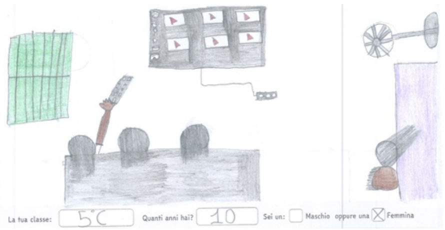

Place represented	Inside of the house	Any room or area clearly inside the house.	I. Inside room, sofa, bed etc.<br>E. Any external place.	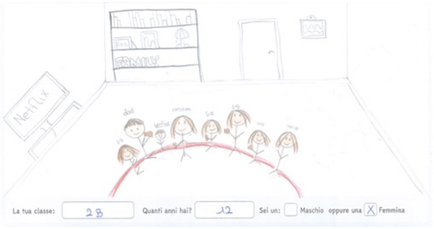 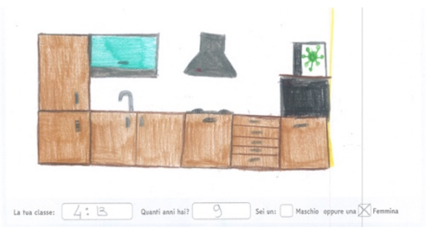
	Surroundings of the house	Any area outside, but in the vicinity of the house.	I. Back or front yard, house surroundings, garden etc.<br>E. Any areas in the house, wider external places.	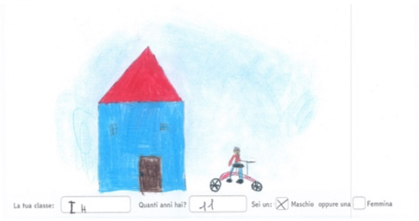 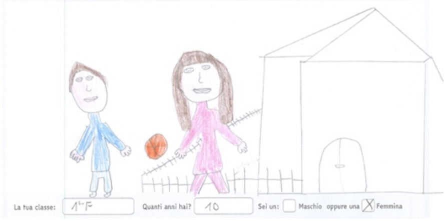
	Other, outdoor or city place	Any external place, natural or urban, that is assumed to be some distance from the house.	I. Beach, parks, mountains, supermarket, etc.	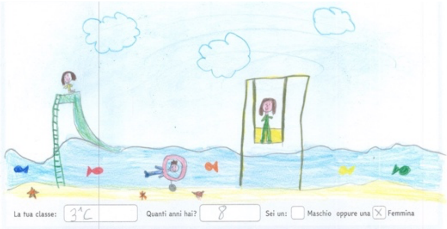 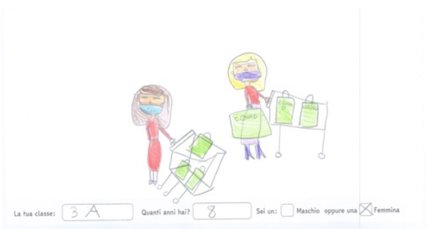

External world visible?	Not visible	There is no view of the outside world.	I. Rooms in the house, places, or furniture.<br>E. Objects or facilities that are assumed to be outside; swimming pools, trampoline, seesaw.	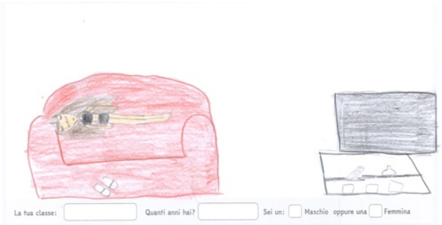
	Partially visible	The outside world is partially visible either through a window or on a portion of the drawing.	I. the following are partially visible: Sky, clouds, sun, moon, garden, playing field, etc.<br>E. the whole scene is external	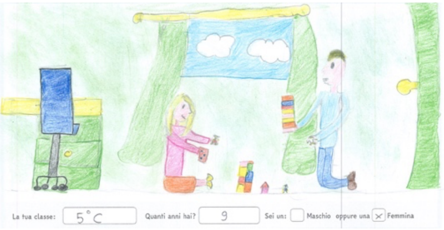
	Most/all of the scene is external	Any external place, natural or urban.	I. Back or front yard, house surroundings, garden, parks, or any other urban place.	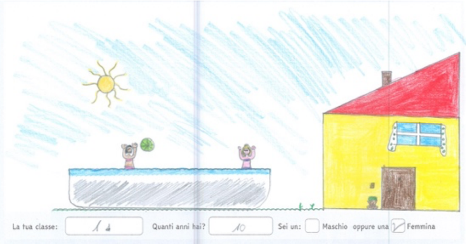

Activity of the main subject	Play, sport	References to recreational or physical activities in all their forms.	I. Group games, outdoor games, table games, children having fun, riding a bicycle, playing with a pet, etc.<br>E. Videogames, screentime.	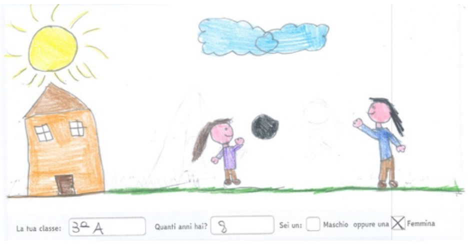 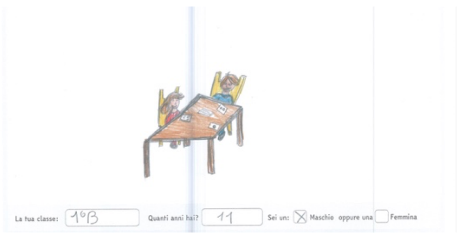
	Personal use of ICT	The subject is using ICT for personal purpose or recreation.	I. Screentime, making a video.<br>E. Studying, homework, distance learning.	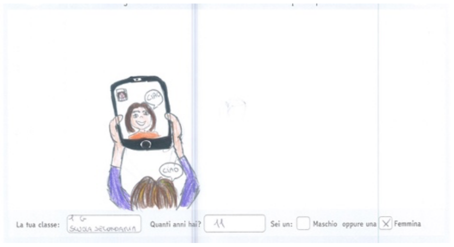
	Daily chores or routines	The subject is doing chores around the house.	I. Helping mom or dad, gardening, tidying the room, cooking, having breakfast<br>E. School homework.	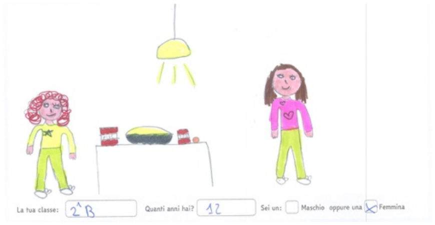
	School or distance learning	The subject is using ICT for school activities or distance learning.	I. Studying, distance learning, classroom video call<br>E. Screentime, personal use of ICT.	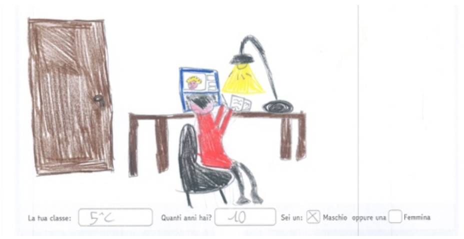

	Escaping the present	All the leisure or recreational activities are aimed at killing time or escaping the present situation.	I. Fantasizing, sleeping, daydreaming.<br>E. Play, screentime.	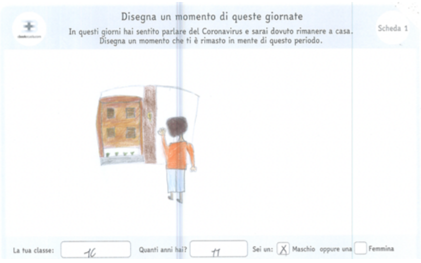 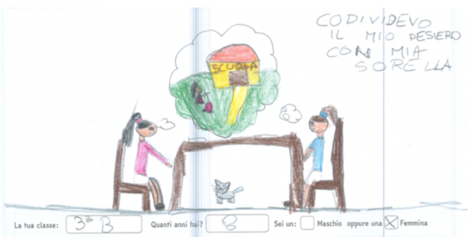

What is represented	Only the main subject (self)	Only the main subject is present in the drawing.	I. only one person is present.<br>E. nobody is present, more than one people are represented.	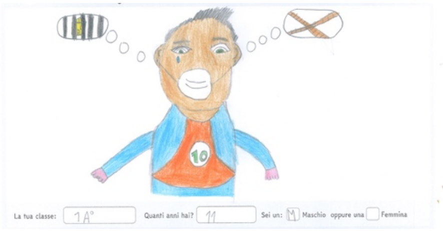
	Main subject and other people	More than one person is present.	I. Peers, friends, siblings, parents or family members, teachers.<br>E. Pets, animals.	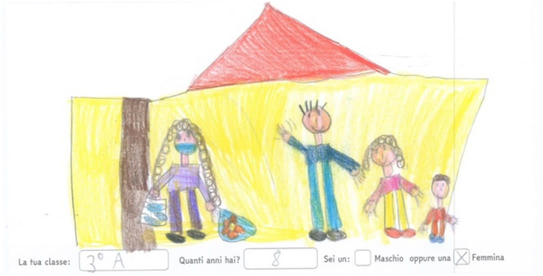
	Only objects, no people	There is no person present in the drawing.	I. Empty rooms, open spaces, desert roads or urban places.<br>E. People on screens.	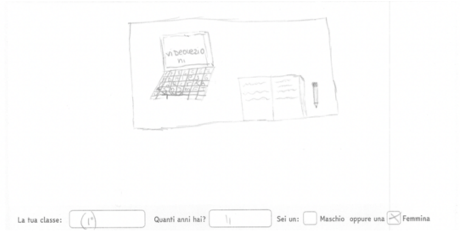
	Pets	Any kind of family pet is represented	I. Any species of pets, mentioning pet names.<br>E. Wildlife.	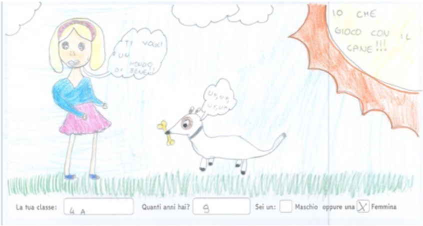 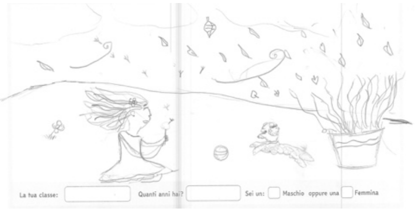

	Covid, death	Representation of the virus, representation of death such as a grave or a coffin.	I. Drawing of the virus, a cemetery, coffin, tomb, or grave.	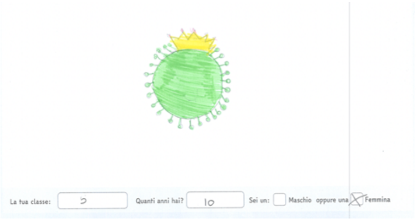 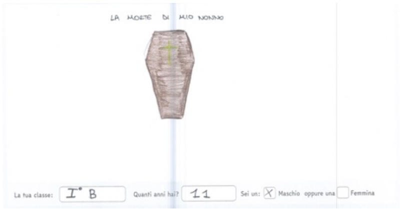

How is ICT represented	Passive use of ICT	Explicit references to the passive use of technology to play or watch videos as entertainment.	I. Videogames, gaming platforms, apps and services that involve watching videos, TV.<br>E. Using devices to have meaningful relationships or to actively work on a project.	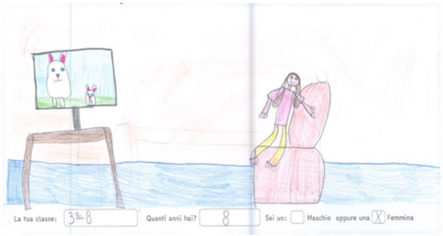 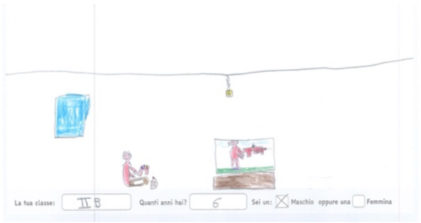
	Only ICT, no people	Some type of ICT equipment is depicted but no people are present.	I. TV or computer screen, videogames, mobile phones.<br>E. Active use o ICT, distance education with other people present.	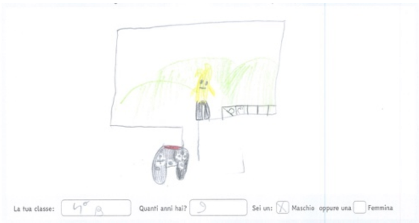
	Active use of ICT	References to the active use of technology to keep in touch with other people, to carry out research or to produce videos for entertainment.	I. Video editing activities, making calls, or sharing information through virtual meeting platforms, using search engines to find information. Typing on the keyboard or using the mouse.<br>E. Video lessons, videogames or watching television.	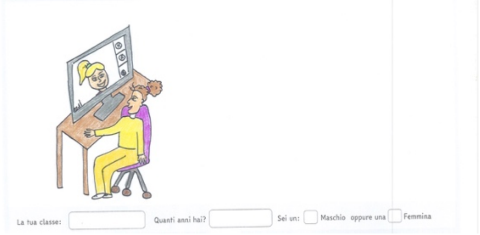
	ICT as part of distance learning	Explicit references to ICT and schools, lessons, homework.	I. The drawings show teachers, assistants, school subjects, school activities.<br>E. Other use of ICT as mentioned above.	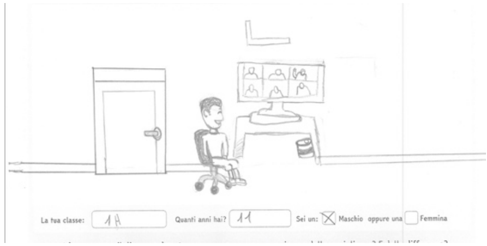
